# A novel role for pigment genes in the stress response in rainbow trout (Oncorhynchus mykiss)

**DOI:** 10.1038/srep28969

**Published:** 2016-07-04

**Authors:** Uniza Wahid Khan, Øyvind Øverli, Patricia M. Hinkle, Farhan Ahmad Pasha, Ida Beitnes Johansen, Ingunn Berget, Patricia I. M. Silva, Silje Kittilsen, Erik Höglund, Stig W. Omholt, Dag Inge Våge

**Affiliations:** 1 Centre for Integrative Genetics, Department of Animal and Aquacultural Sciences, Norwegian University of Life Sciences, NO-1430 Ås, Norway; 2 University of Rochester Medical Center, School of Medicine and Dentistry, Department of Pharmacology and Physiology, Rochester, NY 14642, USA; 3 KAUST catalysis center (KCC), King Abdullah University of Science and Technology, Thuwal 23955, Saudi Arabia; 4 Department of Molecular Biosciences, University of Oslo, NO-0316 Oslo, Norway; 5 Norwegian School of Veterinary Medicine, Institute of Basal Sciences and Aquatic Medicine, NO- 0033 Oslo, Norway; 6 Technical University of Denmark, National Institute of Aquatic Resources, DK-9850 Hirtshals, Denmark; 7 Norwegian Institute for Water Research (NIVA), NO-0349 Oslo, Norway; 8 NTNU - Norwegian University of Science and Technology, NO-7491 Trondheim, Norway

## Abstract

In many vertebrate species visible melanin-based pigmentation patterns correlate with high stress- and disease-resistance, but proximate mechanisms for this trait association remain enigmatic. Here we show that a missense mutation in a classical pigmentation gene, *melanocyte stimulating hormone receptor* (*MC1R*), is strongly associated with distinct differences in steroidogenic *melanocortin 2 receptor (MC2R)* mRNA expression between high- (HR) and low-responsive (LR) rainbow trout (*Oncorhynchus mykiss*). We also show experimentally that cortisol implants increase the expression of *agouti signaling protein* (*ASIP*) mRNA in skin, likely explaining the association between HR-traits and reduced skin melanin patterning. Molecular dynamics simulations predict that melanocortin 2 receptor accessory protein (MRAP), needed for MC2R function, binds differently to the two MC1R variants. Considering that mRNA for MC2R and the MC1R variants are present in head kidney cells, we hypothesized that MC2R activity is modulated in part by different binding affinities of the MC1R variants for MRAP. Experiments in mammalian cells confirmed that trout MRAP interacts with the two trout MC1R variants and MC2R, but failed to detect regulation of MC2R signaling, possibly due to high constitutive *MC1R* activity.

Post-transcriptional processing products of the primordial animal gene proopiomelanocortin (POMC), such as melanocortin hormones and neurotransmitters, are involved in an extensive range of physiological and behavioral functions in vertebrates. Recent pharmacological and genetic studies have focused on the role of the melanocortin system in pigmentation, stress responses, inflammation, energy homeostasis, and sexual function^1^,^2^,^3^,^4^. Identification of selective agonists and antagonists of this system provides potential pharmacotherapies for skin cancer, obesity, and neurodegenerative and inflammatory disease, among others^5^,^6^,^7^. The melanocortin system consists of five distinct seven-transmembrane G protein-coupled receptors (MC1-5R), several POMC-derived agonists and two endogenous antagonists, agouti signaling protein (ASIP) and agouti-related protein (AGRP)^8^,^9^. Furthermore, the small single-pass transmembrane proteins melanocortin receptor accessory protein (MRAP) and its paralog MRAP2 have been shown to provide additional regulation of MCR expression and function^10^,^11^,^12^,^13^. Together these elements form a complex neuroendocrine machinery in which polymorphic genes may show considerable pleiotropy. These effects include adaptive phenotypic diversification of correlated trait clusters (behavioural syndromes, or “animal personalities”) in a number of vertebrate lineages^14^,^15^. A conserved feature is that extensive and distinct melanin-based dermal pigmentation patterns correlate with proactive behavior^15^,^16^,^17^ and high stress- and disease-resistance^18^,^19^.

It is well documented that ectopically expressed agouti and agouti-related proteins interfere with distinct melanocortin receptors^20^,^21^,^22^,^23^. Guided by this, we exploited strains of rainbow trout (Oncorhynchus mykiss) selected for low (low-response, LR) or high (high-response, HR) post-stress cortisol production^24^, previously shown to display correlated behavioral (proactive LR vs reactive HR) and pigment patterns^15^,^25^, to gain deeper insight into the molecular mechanisms linking pigment patterns and behavioral patterns. We show that a missense mutation in melanocortin 1 receptor (MC1R), typically considered a pigmentation gene^26^,^27^ controlling melanin synthesis in skin, is functionally linked with heritable variation in stress resistance. This finding may improve the understanding of the gene-environment interactions underlying individual variation in behavior and physiology, a topic of interest to diverse fields such as evolutionary ecology, population management, animal husbandry, and biomedicine^28^,^29^,^30^,^31^. Understanding the associations between color polymorphisms and other physiological-behavioral trait clusters (coping styles and animal personalities) can be particularly interesting in this context, due to the well recognized role of visual signals in behavioral ecology and evolutionary biology.

## Results

### Sequencing of candidate genes

**Figure 1:**
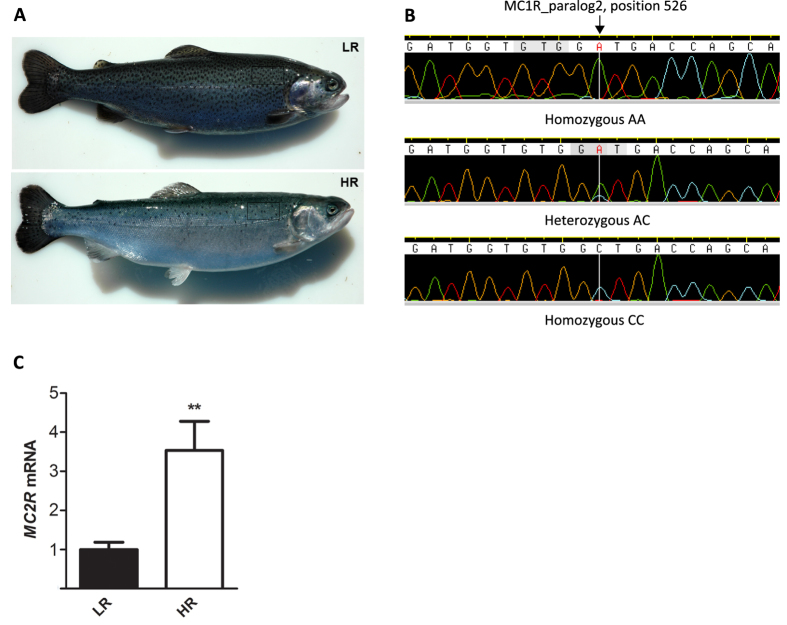
Features distinguishing low-responsive (LR) and high-responsive (HR) fish. (**A**) Representative images of dermal pigmentation in LR (top panel) and HR (lower panel) individuals. (**B**) Sequence chromatograms from homozygous AA (top panel), heterozygous AC (middle panel), and homozygous CC (lower panel) individuals in strain-distinguishing melanocortin 1 receptor (*MC1R*)_paralog2 position 526. Relative expression of (**C**) *melanocortin 2 receptor* (*MC2R*) mRNA in head kidney is increased in high-responsive (HR) (n = 8) compared to low-responsive (LR) (n = 8) fish. mRNA expression levels are presented as fold change normalized to LR average = 1 (mean ± s.e.m; **p < 0.01, two-tailed t-test).

We sequenced the coding region of a number of genes known to be involved in pigmentation and cortisol regulation, including *MC2R*, *MRAP* (FR837908), *MRAP2* (FR837909), *ASIP* (FN821692) and *MC1R*. None of the coding sequences from low-responsive (LR) fish (n = 5) and high-responsive (HR) fish (n = 5) (see Fig. 1A for representative phenotypes) revealed any polymorphism distinguishing the two groups except for *MC1R* (see online supplementary material for details of methods and results). We identified two paralogs, *MC1R*_paralog1 (FN821693) and *MC1R*_paralog 2 (FN821694), each containing an open reading frame encoding a 328 amino acid protein. The *MC1R*_paralog 2 sequence contained a single (non-synonymous) adenine/cytosine (C/A) polymorphism in nucleotide position 526 (c.526C > A), which explicitly distinguished the HR and LR groups (Fig. 1B). All HR individuals genotyped for this polymorphism (n = 13) were homozygous CC, while 10 LR individuals were homozygous AA, and 3 LR individuals were heterozygous AC (Fisher’s exact test for unequal distribution of CC, p < 0.0001). The c.526C > A polymorphism causes a Leu/Met switch in aa-position 176 (L176M), located in the fourth transmembrane domain of the MC1R protein (Figure S1).

### Gene expression studies

Since endogenous ACTH production has been shown to be similar between the LR and HR groups and since the HR steroidogenic response to any given (exogenous) dose of ACTH is approximately twice that of LR^32^, we hypothesized that the MC1R polymorphism might influence cortisol level by interacting with MC2R. In teleosts the head kidney forms the homolog of the mammalian adrenal gland^33^. Therefore, a physical interaction between the two receptors requires MC1R expression in the head kidney. A real competitive (rc)PCR in 8 HR and 8 LR fish confirmed that MC1R was expressed in head kidney of both groups (all logEC_50_ values >−19). Expression of MC1R in head kidney has also recently been shown in zebrafish^34^.

Using Chinese hamster ovary (CHO) cells, it was showed that ACTH caused a strong MC2R-mediated cAMP response when *MC2R* was co-transfected with *MRAP*, while cells co-transfected with *MC2R* and *MRAP2* only showed a small increase in cAMP^13^,^35^. Guided by this, we found in a pooled group of 8 HR and 8 LR individuals that *MC2R* and *MRAP* mRNA levels were strongly correlated (R^2^ = 0.67, p < 0.001), while *MC2R* and *MRAP2* mRNA levels were not correlated (Figure S2). This suggests that major features of the *MRAP/MRAP2/MC2R* regulatory architecture seen in mice and humans are similar in teleost fishes, and that the mRNA level of *MC2R* in the head kidney tissue reflects the level of MC2R-MRAP signaling. Assuming this, the observed difference in *MC2R* mRNA levels between the two groups (Fig. 1C) implies that MC2R-MRAP signaling function is lower in LR than in HR individuals under baseline conditions. Since MRAP has the ability to also bind MC1R^11^,^36^, we hypothesized that this protein could represent a molecular link between the MC1R-polymorphism and MC2R signalling.

### Homology modelling and protein-protein docking

As an initial test of this hypothesis we performed a molecular dynamics study using homology modelling and protein-protein docking to examine a possible interaction between a MRAP dimer and the two MC1R variants. MRAP forms a stable, antiparallel homodimer^37^. Several MC1R structures were generated by I-TASSER, and the energetically most stable structure (Figure S1) was used for MC1R – MRAP docking studies. The ClusPro online server generated several dimeric models for MRAP, and a perfect antiparallel low volume model (Figure S3) was selected for the MC1R-MRAP docking study.

**Figure 2:**
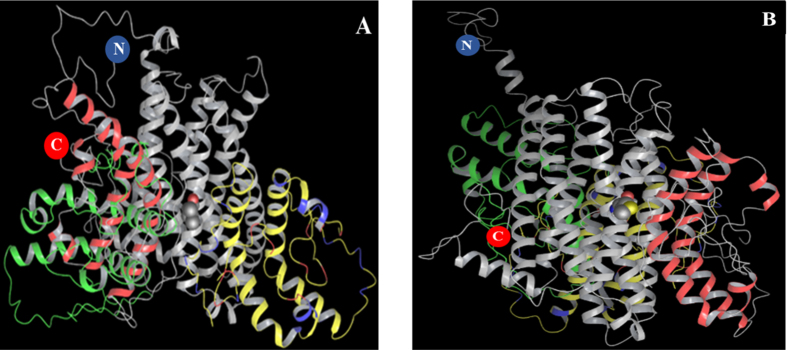
The 3 least energy docking poses for MRAP-dimer are shown for (**A**) (MC1R-176Leu) and (**B**) (MC1R-176Met), respectively. The MCIR helices are displayed in grey, and the N-terminal and C-terminal ends are indicated in blue and red, respectively. The MRAP dimers are colored according to their energy sequence red < yellow < green. This indicates the most probable complex is MC1R (grey) and MRAP dimer (red) and second most probable complex will be MC1R (grey) and MRAP dimer (yellow) while third most probable complex will be MC1R (grey) and MRAP dimer (green).

The three least energy docking poses for this MRAP dimer are shown in Fig. 2. The MC1R-176Leu variant and lowest energy MRAP (red) complex indicates that the nearest MRAP residue (41A) is located within 3.9 Å, while the MC1R-176Met variant and lowest energy MRAP (red) complex has the nearest MRAP residue (17P) located within 4.8 Å. In general, MRAP binds close to MC1R residue 176. A close interaction (<2.0 Å) between MRAP dimer and the MC1R-residues Thr5, Gln7, Tyr81 and Thr169 was observed in this binding mode. In the case of MC1R-176Met (Fig. 2B), the least energy dock pose (red) is close to residue 176Met and the orientation is parallel to MC1R helix 4. This binding mode shows close interaction (<2.0 Å) between MRAP and MC1R-residues Arg166, Thr169, Val187, Tyr188 and Arg225. These results indicate that the MC1R-176Leu → Met change could affect MC1R-MRAP binding.

### Mammalian cell experiments

Given the above theoretical results, and the fact that both MC1R and MC2R are expressed in head kidney, we then explored possible interaction targets for these two receptors in HEK293 cells, a classical model for evaluating G protein-coupled receptor signaling. MC2 receptor accessory protein (MRAP) is a prerequisite for MC2R function, and all five human MC receptors interact with the 172 aa human MRAPα *in vitro*^11^. We therefore tested whether 176Leu or 176Met MC1 receptors interacted with trout MRAP expressed in the same cells by precipitating HA-tagged receptors and blotting for Flag-tagged MRAP and vice versa (Fig, 3A). Both variants of trout MC1 receptor co-precipitated with the 78 aa trout MRAP, although not quantitatively.

**Figure 3:**
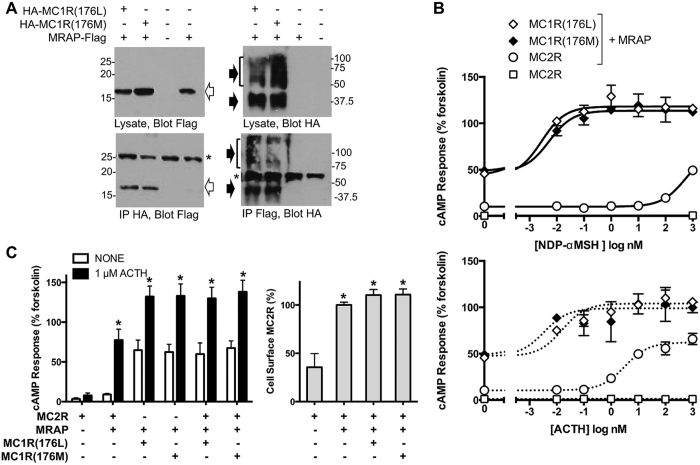
Analysis of trout MC receptor and MRAP *in vitro*. (**A**) Co-precipitation of MRAP and MC1 receptors. Cells were transfected with HA-tagged trout MC1 receptors and Flag-tagged trout MRAP as shown. Cell lysates were immunoprecipitated (IP) with anti-HA or anti-Flag antibodies and lysates and immunoprecipitates run on SDS-PAGE and immunoblotted with anti-HA or anti-Flag antibodies. Immunoprecipitate gels (bottom panels) were loaded with 5x more per lane than lysate gels (top panels). Asterisks denote IgG chains, open arrows MRAP-Flag, and solid arrows HA-MC1 receptors. The single MRAP band is consistent with the lack of a predicted N-glycosylation site. Trout MC1Rs, with multiple potential glycosylation sites, ran in broad bands near the expected MW of nonglycosylated and core glycosylated receptor, and in diffuse high MW bands typical of heterogeneously glycosylated mature receptor. (**B**) Agonist specificity of MC1 and MC2 receptors. Cells expressing the cAMP reporter CRE-luciferase and MC receptors with or without MRAP were stimulated with NDP-αMSH or ACTH. Mean ± range of duplicates is shown. (**C**) Effect of MC1 receptors on function and expression of MC2 receptors. (Left) Cells were transfected as shown and cAMP responses determined with or without 1 μM ACTH. Results shown are mean ± SEM from 7–9 experiments, each in duplicate. (Right) Cells were transfected and the relative expression of HA-MC2R on the cell surface was measured by ELISA; only the MC2 receptor was HA-tagged. *P < 0.05 vs. no ACTH (left) or MC2R alone (right).

Having verified that trout MC1R and MC2R can bind MRAP, we tested a model where the two receptors could compete for MRAP. If the MC1R-176Met variant binds more MRAP than MC1R-176Leu, that would reduce the availability of MRAP for the MC2R, possibly resulting in a reduced response to ACTH. Like MC1 receptors of other species, both variants of trout MC1 receptors displayed very strong constitutive activity and additional responses to the potent agonist NDP-α-MSH and ACTH (Fig. 3B, diamonds). MC1R signaling was the same for 176Met and 176Leu variants and not substantially affected by MRAP (not shown). As expected, signaling by trout MC2 receptors was highly dependent on trout MRAP. Trout MC2R showed >50-fold increase in cAMP-reporter activity in response to ACTH (EC_50_ = 3.4 nM) and low levels of constitutive activity and NDP-α-MSH responsivity (Fig. 3B, open symbols).

In similar experiments, EC_50_ values for ACTH were 0.02 and 17 nM for human MC2R expressed with human MRAPα and trout MRAP, respectively, versus 320 and 8.4 nM for trout MC2R expressed with human MRAPα and trout MRAP. It is likely that the fish MRAP/fish MC2R complex achieves a conformation with high affinity for ACTH based on the 40-fold lower EC_50_ value for fish MC2 receptor/fish MRAP compared to fish MC2 receptor/mammalian MRAP. Regions of MRAP critical for activity are similar in fish and mammals and present even in the short trout MRAP sequence. In particular, the activity of rainbow trout MRAP is greatly reduced by mutation of trout MRAP residues 12–15 from YDYL to AAAA; alanine substitution of the corresponding residues in mouse MRAP (LDYI) is likewise inactivating^35^,^38^. Unfortunately, the high constitutive activity of MC1R impeded the assessment of how the two trout MC1 receptor variants impact signaling by the trout MC2 receptor. The constitutive activities of 176Leu and 176Met MC1R were indistinguishable and as strong as the ACTH-stimulated response of MC2 receptors (Fig. 3C, left). Furthermore, in cells expressing both MC1R and MC2R, the ACTH-stimulated increase in cAMP was much lower than that expected if the responses were simply additive. The amount of HA-tagged trout MC2R on the cell surface was increased by trout MRAP, whereas co-expression of MC1R variants had no significant effect (Fig. 3C, right). MC2 receptor responses could not be isolated in additional experiments using different levels of receptors, MRAP and ACTH (data not shown).

### Cortisol implants in LR – fish

Since there was no significant difference in signaling between either the constitutive or agonist- stimulated activity of the 176Leu and 176Met variants of MC1R, the difference in pigmentation between LR and HR fish cannot be directly attributed to the MC1R-176Met/Leu variants. An alternative model could be that MC1R signaling in HR-fish is downregulated by higher concentrations of the inverse antagonist ASIP in the skin, since this gene could be influenced by a higher cortisol level, analogous to how glucocorticoids influence the expression of *ASIP* in human adipocytes^39^. To test this possibility, LR individuals (n = 8) were provided with cortisol implants for two weeks and compared with sham-treated LR controls (n = 8). This treatment yielded an elevation of plasma cortisol from 26.0 (±5.9) ng/ml in sham-treated controls to 192.7 (±21.8) ng/ml in the treated group (t = 7.39, df = 14, p < 0.001). In this group of fish (which was about one year older than the original test fish), *ASIP* mRNA expression was below the detection limit of real competitive PCR in all sham-treated fish (i.e. all log_10_ (EC_50_) < −19)^40^ (Table 1), while *ASIP* mRNA expression was well into the detectable range in all 8 cortisol treated fish (all log_10_ (EC_50_) >−14) (Chi-Sq = 12.25, p < 0.001) (implying at least 10^4^ higher concentration of *ASIP* mRNA in the treated groups). The cortisol treatment had no effect on *MC2R* mRNA expression in head kidney (t = 1.09, df = 14, p = 0.30).

### Characterization of “spotted” and “non-spotted” fish outside the LH/HR selection lines

**Figure 4:**
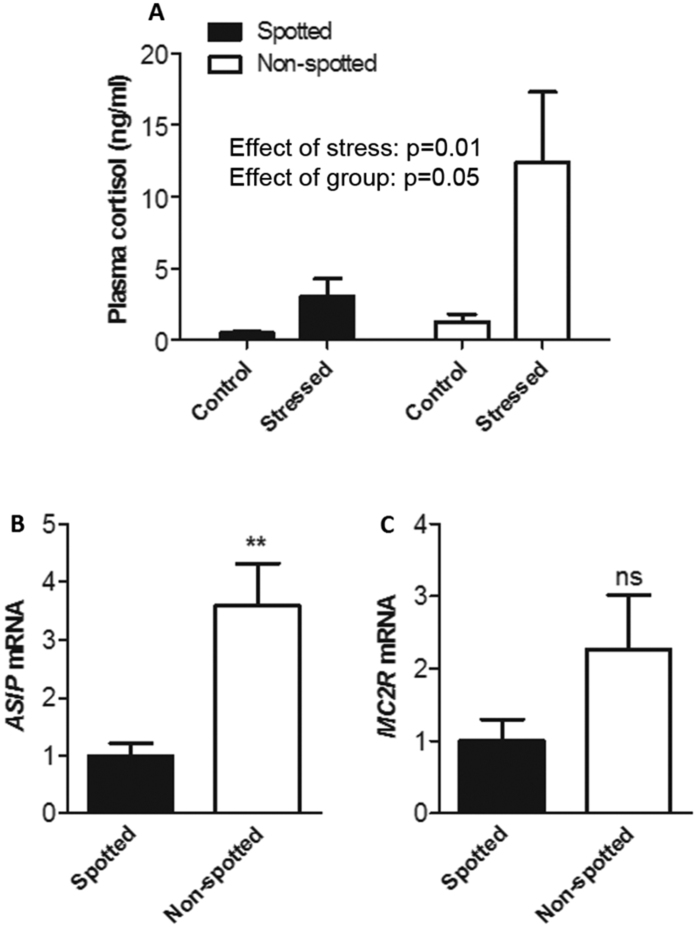
Molecular characterization of an arbitrarily chosen population of rainbow trout. Two-tailed t-test is used for group comparisons unless otherwise stated. (**A**) Plasma cortisol levels of acutely stressed fish (n = 8 + 8) and non-stressed controls (n = 8 + 8). Two-way ANOVA statistics are given on graph. (**B**) *Agouti-signaling protein* (*ASIP*) mRNA abundance in skin of non-stressed fish (n = 8 + 8, mean ± s.e.m.; **p < 0.01). (**C**) Melanocortin 2 receptor (*MC2R*) mRNA abundance in head kidneys of non-stressed fish (n = 8 + 8, mean ± s.e.m., n.s., p = 0.14).

To rule out possible artifacts stemming from the HR-LR selection regime, we repeated our study with 200 rainbow trout obtained from an arbitrarily chosen aquaculture producer in Norway (the HR-LR lines originated in the UK). These fish were photographed and analyzed for variable eumelanin pigmentation^15^. Two groups containing the most spotted (“spotted”) and the least spotted fish (“non-spotted”) were chosen for further study. Similar to the HR/LR-fish, there was a significant difference in plasma cortisol between the two groups (Fig. 4A), and a significant difference in skin *ASIP* mRNA expression was seen in undisturbed control fish (Fig. 4B) as well as in *MC2R* mRNA expression in head kidney (Fig. 4C). Ten non-spotted fish were homozygous CC, while 3 spotted fish were homozygous AA and 7 were heterozygous AC (p < 0.001).

## Discussion

In the present study we revealed that a sequence variant (c.526C > A ) in the MC1R_paralog 2 gene segregated consistently in two strains of rainbow trout (*Oncorhynchus mykiss*), selected for low (low-response, LR) or high (high-response, HR) post-stress cortisol production. These strains have previously been shown to display correlated behavioral (proactive LR vs reactive HR) and pigmentation patterns^15^. Our results show that a missense mutation in a classical pigmentation gene (*MC1R*) is strongly associated with variable MC2R transcription and downstream effects in the HPA axis. Therefore, the increased cortisol response to ACTH shown by HR fish relative to LR fish^32^ is likely explained by an increased MC2R function in the HR fish (Fig. 1C).

**Figure 5:**
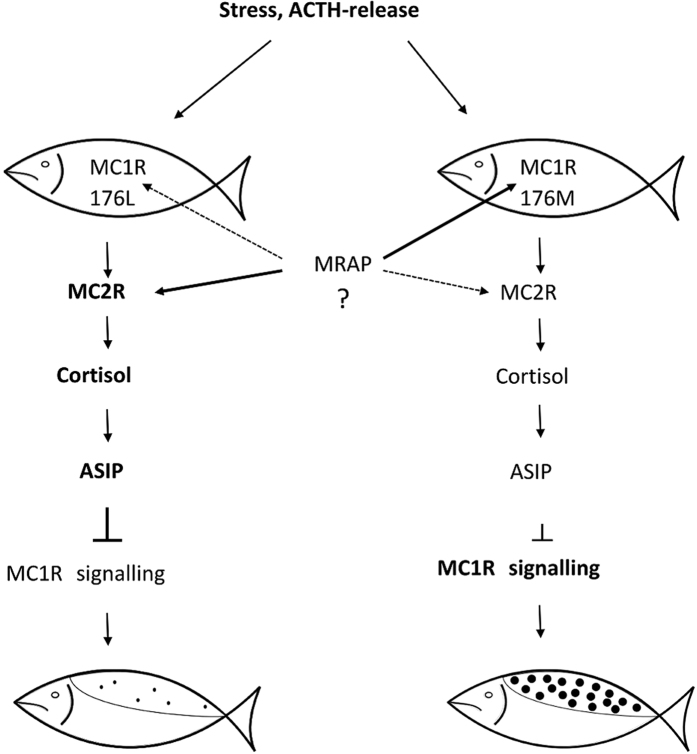
The proposed model of stress response in high-responsive (MC1R-176L) and low-responsive (MC1R-176M) fish. In the left column (MC1R-176L) MC2R is highly responsive to ACTH released under stress, which in turn increases the production of cortisol. Higher plasma cortisol levels increase ASIP expression in skin. ASIP is known to be an inverse agonist of MC1R, causing lower MC1R signalling and lower production of black pigment (few and small black skin spots). In the right column, we speculate that MRAP binds more MC1R due to the 176M-variant, reducing the amount of MRAP available to interact with MC2R and reducing MC2R signaling. This gives a lower cortisol response, lower ASIP expression and stronger MC1R signalling, since the amount of inverse agonist (ASIP) is reduced. Stronger MC1R-signalling induces production of black pigment (many and larger black skin spots).

These results are not likely to be an artefact of the HR-LR selection program^24^, since entirely similar patterns were seen in a outbred control population of rainbow trout. How can then a conservative amino acid change (L176M) in MC1R possibly influence MC2R expression? MC2R mRNA is regulated by its ligand ACTH in rainbow trout, similar to what is found in other vertebrates^41^, but there is no antibody that allows quantification of MC2 receptor protein in tissue. Melanocortin 2 receptor accessory protein (MRAP) is a key protein for MC2R membrane expression and ACTH binding, which also has the ability to bind MC1R, demonstrated by co-precipitation of MC1R-MRAP in this and other studies^11^. Based on our initial results and available literature we hypothesized that the *MC1R* is expressed in the head kidney where the MC1R-176 Met/Met variant may bind more avidly than the MC1R-176 Leu/Leu to the MRAP protein. This might reduce the amount of MRAP available for MC2R and consequently reduce responsiveness to ACTH and thus cortisol production (Fig. 5). An exclusive prediction from this conceptual model is that *MC1R* is expressed in the head kidney in both HR and LR fish, which was confirmed in this study.

We further used homology modelling and protein-protein docking to investigate if the L176M shift possibly could influence MC1R-MRAP binding. The position of the L176M polymorphism in the fourth transmembrane domain is compatible with previous reports implicating transmembrane domains of MRAP in MC2 receptor signalling^11^,^42^,^43^. Our docking results also indicate that MRAP dimer may bind close to the MC1R-176 position, and that the 176Leu → Met change may affect the MRAP-MC1R binding (Fig. 2).

To validate the model in a biological assay we used a standard model for analyzing GPCR function, HEK293 cells, to test if the MC1R-176Met variant binds more MRAP than MC1R-176Leu. In these overexpression studies, both trout MC1 receptor variants displayed very strong constitutive activity (Fig. 3B, diamonds), which made it impossible to measure any differences in MC2R-specific signalling attributable to MC1R-176L and MC1R-176M, respectively. Expression of MC2 receptor on the cell surface was not significantly altered by MC1R (Fig. 3C, right). It is therefore hard to see how ACTH can regulate MC2 receptors in the face of the high cAMP concentrations generated by constitutively active MC1 receptors if MC1 and MC2 receptors populate the same cells in the head kidney. However, the levels of receptor proteins *in vivo* are unknown and possibly dramatically lower than those reached in transfected HEK293 cells. It is also possible that factors such as ASIP reduce MC1R activity *in vivo*.

Since the two MC1R variants behave almost identically with respect to both constitutive activation and response to NDP-α-MSH, the differences in skin melanin spots could not be directly attributed to the MC1R polymorphism. We therefore speculated that the skin pigmentation difference could be an indirect effect of lower plasma cortisol levels over time in LR-type (i.e. Met/Met and Met/Leu) individuals. It has previously been shown that glucocorticoids influence the expression of *ASIP* in human adipocytes^39^. LR-fish supplied with cortisol implants for two weeks showed a significantly higher *ASIP* expression compared to controls. Different *ASIP* expression was also seen when an arbitrarily chosen group of rainbow trout were phenotypically grouped into LR and HR type and compared (Fig. 4B). The higher amount of agouti protein in the skin will efficiently antagonize the MC1 receptor, and less black eumelanin will be produced in the HR-fish. The observed correlation between pigmentation and cortisol level^15^ is more likely to be a downstream effect of increased cortisol than a direct effect of the pigment gene variant.

We have shown how a phenotypic correlation between stress-coping traits and skin pigmentation^15^ is strongly associated with a single nucleotide polymorphism in a classical pigmentation gene, MC1R. The results suggest that the divergent HR-LR selection for post-stress cortisol levels has heavily exploited a naturally existing pleiotropism causing a negative correlation between corticosteroid based stress responses and skin melanism.

## Methods

### Fish

High- (HR) and low-responsive (LR) trout lines were created by selection for divergent post-stress cortisol levels^24^. These lines are established as a comparative model for heritable variation in stress coping style^25^,^44^,^45^, and their behavior and physiology have been well described. All experimental protocols of the experimental animals were reviewed and approved in advance by the Norwegian Animal Research Authority (NARA). The methods were carried out in accordance with the Animal Welfare Act and the Regulation of Animal Experiments in Norway.

### Gene sequencing and expression studies

Molecular studies consisted of amplification, sequencing and SNP detection of the coding region of *MC1R* (paralog 1 and 2), *MC2R*, *ASIP*, *MRAP* and *MRAP2* (detailed methods in online supplementary material). Sequences were aligned and screened for SNPs using the programs phred, phrap and consed^46^,^47^. Real competitive PCR (rcPCR)^40^ was used to study the tissue- and allele-specific expression of *MC1R*, *MC2R* and *ASIP* mRNA, while real time PCR quantification^48^ was used for head kidney expression of *MRAP*, *MRAP2* and *MC2R* in correlation studies. The effect of cortisol on *ASIP* expression was studied in adult LR fish transferred to social isolation and provided with cortisol implants (84mg/kg body weight) in the peritoneal cavity. After 14 days with implants, samples were collected for gene expression analysis using rcPCR.

Studies to confirm the occurrence and role of the identified *MC1R* polymorphism outside the HR-LR selection regime were performed using two hundred rainbow trout from a commercial Norwegian breeder. Quantification of melanisation and behavioral analysis was carried out as described by Kittilsen *et al*.^15^. The 16 most spotted and the 16 least spotted individuals were selected for further studies, including behavior and plasma cortisol concentrations (analyzed following Sorensen *et al*.^49^) in both stressed fish and non-disturbed isolated controls. Gene sequencing and tissue specific expression analysis (based on samples from non-stressed fish) was carried out as above.

### Statistical analysis

Unless otherwise specified, all bars show means ± S.E.M. The statistical test includes two-tailed student t-test and two-way ANOVA. Normality and variance homogeneity was checked by Kolmogorov-Smirnov and Levene’s test, respectively. Behavioral data were square-root transformed in order to conform to criteria for parametric statistics. Correlations were analyzed statistically by linear regression. Further details of rcPCR design and statistical analysis are given in online extended methods section.

### Homology models and protein – protein docking

Homology models were constructed for the MC1R variants 176Leu (CBL93117) and 176Met (CBL93118) as well as for MRAP (CCA30387), using the online server I-TASSER^50^,^51^. I-TASSER uses several templates in the protein databank (RCSB) for the prediction of homology of protein structure. In order to estimate the stability of homology models of both the mutants, molecular dynamics simulation was performed. Each protein was solvated in TIP3 water box and a water layer was kept on each side (10 Å) to avoid periodic overlap while electrostatic cutoff was set at 9 Å. The simulation time was 50 ns and the temperature was 300 K. During simulation 50000 snapshots were taken at regular intervals. All calculations were performed using Desmond software with OPLS force field^52^. The MRAP dimer modelling was performed using the ClusPro online server^53^,^54^,^55^. Docking of the MRAP dimer into MC1R variants was also performed using the ClusPro online server.

### Reagents for cell studies

Plasmids encoding trout MC receptors and MRAP were synthesized by Genewiz. Constructs encoding 176Leu and 176Met variants of paralog 2 of the trout MC1 receptor were designed with triple HA tags on the amino-terminus and trout MC2 receptor with a single N-terminal HA tag. All receptors were cloned into pcDNA3.1. MC1 receptors lacking tags were prepared using QuikChange from Stratagene. Trout MRAP was cloned into a pCI-Neo vector that introduced a triple Flag tag at the C-terminus. hACTH (1–24) was from Phoenix Peptides.

### cAMP Responses

Human embryonic kidney (HEK293) cells were maintained in DMEM with 5% fetal bovine serum at 37 °C in 5% CO_2_−95% air. To measure cAMP responses, cells were seeded in white 96 well plates, grown for 24 or 48 h, and transiently transfected with 50 ng DNA/well using Lipofectamine 2000, typically with equal amounts of plasmids encoding MC receptor(s), MRAP and the cAMP reporter CRE-luciferase^56^. DNA concentrations were balanced with empty vector or plasmid encoding GFP. The day after transfection, cells were incubated in DMEM containing 0.1% BSA and peptides or 20 μM forskolin for 4 to 6 h when media was removed and cells lysed in 50 μl/well Firefly Luciferase Assay Reagent from Nanolight. Luminescence was quantified in a BioTek platereader and responses expressed as percent of the forskolin response.

### Plasma Membrane Expression of Receptors and MRAP

A previously described modified ELISA protocol was used to quantify MC2 receptor on the surface of nonpermeabilized cells^57^. In brief, cells grown in 24 well poly-L-lysine-coated dishes were transfected with 250 ng DNA/well. After 24 or 48 h, cells were washed, fixed for 20 min with 3% paraformaldehyde in PBS, washed again and incubated in PBS containing 5% nonfat dry milk and monoclonal anti-HA antibody at 1:1000 (Covance HA-11) for 1 h at room temperature. Plates were washed twice and incubated with 1:5000 HRP-labeled anti-mouse IgG for 1 h, washed and incubated with TMB (3,3′,5,5′-tetramethylbenzidine) peroxidase substrate until color developed when 200 μl of 10% sulfuric acid was added to stop the reaction and 300 μl was transferred to a 96 well plate and absorbance at 450 nm measured.

### Immunoprecipitation and Immunoblotting

Cells in 35 mm plates were transfected with 1 μg DNA, washed, and lysed in 0.25 ml lysis buffer (150 mM NaCl, 50 mM Tris, 1 mM EDTA, 0.1% n-dodecylmaltoside, pH 8.0 with protease inhibitors). Lysates were centrifuged at 10,000 × g for 20 min at 4 °C. Samples of the supernatant fractions were saved for analysis of total lysate and the remaining solution was tumbled overnight at 4 °C with either 25 μl of M2-anti-Flag antibody immobilized on agarose beads (Pierce) or with anti-HA antibody at 1:1000. Anti-HA immunoprecipitates were collected on 25 μl of protein G immobilized on magnetic beads (Millipore), washed and mixed with 4x LDS NuPage sample buffer containing 200 mM dithiothreitol. Samples of lysates and immunoprecipitates were run on 8–16% PAGEr gels from Lonza, transferred to nitrocellulose, and blotted overnight at 4 °C with 1:5000 dilutions of monoclonal anti-HA or M2 anti-Flag antibodies in TBS-T buffer containing 5% milk. Blots were washed and incubated with 1:5000 HRP-labeled anti-mouse IgG and proteins visualized by standard protocols.

## Additional Information

**How to cite this article**: Khan, U. W. *et al*. A novel role for pigment genes in the stress response in rainbow trout (Oncorhynchus mykiss). *Sci. Rep.* **6**, 28969; doi: 10.1038/srep28969 (2016).

## Supplementary Material

Supplementary Information

## Figures and Tables

**Table 1:** Expression of Agouti Signaling Peptide (ASIP) in rainbow trout skin analyzed after 14 days with cortisol implants.

Treatment	Individual no.	S18	β-actin	ASIP
Vehicle	1	−10.21	−11.85	−22.83
Vehicle	2	−10.17	−11.76	−20.85
Vehicle	3	−10.31	−11.72	<−23
Vehicle	4	−10.36	−11.85	<−23
Vehicle	5	−10.19	−12.00	−22.49
Vehicle	6	−10.13	−12.06	<−23
Vehicle	7	−10.11	−11.96	−21.96
Vehicle	8	−10.07	−12.08	<−23
Cortisol	1	−10.09	−11.62	−13.75
Cortisol	2	−10.15	−11.60	−13.57
Cortisol	3	−10.20	−11.66	−13.71
Cortisol	4	−10.12	−11.40	−14.14
Cortisol	5	−10.28	−11.69	−14.03
Cortisol	6	−10.16	−11.66	−13.95
Cortisol	7	−10.17	−11.70	−14.04
Cortisol	8	−10.15	−11.62	−13.95

log EC_50_ values from real competitive PCR are given. *ASIP* expression was below detection limit for reliable quantification using rcPCR in n = 8 untreated controls (log EC_50_ < −19), but clearly detectable in n = 8 cortisol treated fish (Chi-Sq = 12.25, p < 0.001). No such effect was seen on two reference genes S18 and β-actin.
