# Supplementary material for: A novel role for pigment genes in the stress response in rainbow trout (Oncorhynchus mykiss)
Source: Sci Rep. 2016 Jul 4;6:28969. doi: 10.1038/srep28969 (PMC4931468; doi:10.1038/srep28969)
Supplement: Supplementary Information [file srep28969-s1.pdf]

SUPPLEMENTARY INFORMATION

A novel role for pigment genes in the stress response in rainbow trout (Oncorhynchus mykiss).

Uniza Wahid Khan[1], Øyvind Øverli[1], Patricia M. Hinkle[2], Farhan Ahmad Pasha[3], Ida Beitnes Johansen[4], Ingunn Berget[1], Patricia I.M. Silva[1], Silje Kittilsen[5], Erik Höglund[6,7], Stig W. Omholt[8], Dag Inge Våge[1,*]

1 Centre for Integrative Genetics, Department of Animal and Aquacultural Sciences, Norwegian University of Life Sciences, NO-1430 Ås, Norway. 2 University of Rochester Medical Center, School of Medicine and Dentistry, Department of Pharmacology and Physiology, Rochester, NY 14642, USA. 3 KAUST catalysis center (KCC), King Abdullah University of Science and Technology, Thuwal 23955, Saudi Arabia. 4 Department of Molecular Biosciences, University of Oslo, NO-0316 Oslo, Norway. 5 Norwegian School of Veterinary Medicine, Institute of basal sciences and aquatic medicine, NO- 0033 Oslo, Norway. 6 Technical University of Denmark, National Institute of Aquatic Resources, DK-9850 Hirtshals, Denmark. 7 Norwegian Institute for Water Research (NIVA), NO-0349 Oslo, Norway. 8 NTNU - Norwegian University of Science and Technology, NO-7491 Trondheim, Norway.

* to whom correspondence should be addressed. E-mail:

daginge.vage@nmbu.no

This file contains Supplementary text, Supplementary Figures 1-3 with legends and Supplementary Tables 1-4.

Supplementary Methods:

Project structure and fish populations

During the course of this project, five separate studies were carried out in the following sequence: 1) Rainbow trout (*Oncorhynchus mykiss)* strains bred for divergent post-stress cortisol levels (high responsive, HR, and low responsive, LR) [1] were used for initial sequencing of the *O. mykiss MC1R*, *MC2R* and *ASIP* genes. Five HR and five LR fish of the 4th generation were used. 2) Eight HR and eight LR fish of the 5th generation were sampled to confirm the non-random distribution of c.526C>A. These fish were also used to exclude line-distinguishing polymorphisms in other candidate genes, and investigate expression patterns of *MC1R*, *MC2R*, and *ASIP* by real competitive PCR. 3) Sixteen LR fish of the 5th generation were transferred to rearing in social isolation, eight of which were subsequently treated with exogenous cortisol to test the hypothesis that corticosteroid exposure elevates *ASIP* mRNA expression in skin. 4) *MRAP* and MRAP2 were sequenced from head kidney tissue from an additional 8 LR and 8 HR fish (as a limited amount of tissue was available from the initial fish) and expression of these genes was analyzed by real-time PCR (chosen here over real competitive PCR as paralog specific expression analysis was no longer required). 5) Finally, association of the c.526C>A polymorphism with behavior, pigmentation, and physiological characteristics typical of the HR-LR contrast lines were confirmed in spotted and non-spotted fish from an arbitrarily chosen commercial breeder in Norway. This study population was included to rule out possible artifacts (e.g. founder effects, genetic drift) due to the HR-LR selection regime

Gene sequencing

MC1R: cDNA was initially denatured for 10 min at 95 ^oC and thereafter amplified for 40 cycles at 95 ^oC for 30 sec, 59 or 60 ^oC for 30 sec (depending on T_m for primers), 72 ^oC for 1 min, using AmpliTaq Gold® (Applied Biosystems). Amplification primers (Suppl. Table 1) 7600 and 7601 were designed based on available rainbow trout ESTs (TC152435, TC152435). The resulting fragments were isolated from the PCR reaction mix using Illustra GFX™ PCR DNA and Gel Band Purification Kit (GE Healthcare). The isolated fragments were then processed for sequencing using the BigDye® Terminator v3.1 kit (Applied Biosystems) with individual primers and analyzed using 3730 DNA-analyzer (Applied Biosystems). A partial cDNA sequence of *MC1R* obtained from the above sequencing was used to design the gene specific primers for Rapid Amplification of cDNA Ends (RACE), 7613 and 7614 for 3'-RACE and 7621 and 7622 for 5'-RACE, respectively (Suppl. Table 1). First Choice RLM-Race kit (Ambion) was used for amplification of cDNA from skin tissue in order to identify the 5' and 3' end sequences. After reverse transcription, a two-step PCR amplification for 5' and 3' ends of RNA was carried out as described by the manufacturer. We did not succeed in amplifying the 3' end using an RLM-race kit, so a Smart Race cDNA Amplification Kit (Clontech) was used instead. Total RNA was reverse transcribed to 3′-RACE ready cDNA, according to the manufacturer's instructions. In brief, trout *MC1R*-specific primers 3′-Smart race primers (7635 and 7636, Suppl. Table 1), were designed based on the sequence information of the trout *MC1R* and used in combination with a universal primer mix (UPM) for 3′-RACE.

Touchdown PCR was performed using Advantage 2 PCR kit (Clontech). The reaction mix includes 2.5 µl of RACE-ready cDNA, 10 pmol of gene-specific primers, 30 pmol of UPM, 0.2

μM of dNTP, 1× Advantage 2 Taq polymerase and Advantage 2 PCR buffer in a final volume of 50 μl. The touchdown PCR protocol consisted of the following: 1) an initial denaturation of 1 min at 94 °C; 2) five cycles of 30 sec at 94 °C and 3 min at 72 °C; 3) five cycles of 30 sec at 94 °C, 30 sec at 70 °C, and 3 min at 72 °C; and 4) 40 cycles of 30 sec at 94 °C, 30 sec at 68 °C, and 3 min at 72 °C. The 3′-RACE PCR yielded a product of approximately 650 bp. Amplified PCR products were purified with Montage™ PCR centrifugal filter devices (Millipore). Purified fragments were cloned into pGEM-T easy vector system and -transformed into E.coli (XL-blue) competent cells (Promega). QIAprep Spin Miniprep Kit (Qiagen) was used for isolation of recombinant plasmid DNA. Sequencing was performed with BigDye® Terminator v3.1 kit (Applied Biosystems), using primers 7627 and 7628.

***ASIP* :** To sequence the full CDS for *ASIP*, the primers (Suppl. Table 1), 7663 and 7664 were designed using an EST sequence available at NCBI (BX309917.3). The PCR protocol consisted of the following: 1) an initial denaturation of 2 min at 92 °C; 2) 45 cycles of 30 sec at 92 °C, 30 sec at 56 °C, and 1 min at 68 C. The PCR product was purified and sequenced by BigDye® Terminator v3.1 kit (Applied Biosystems), using 7663 and 7664 primers. In this case Herculase (Agilent Technologies) was used for amplification.

MRAP and MRAP2: For amplifying *MRAP*, the primers 7806 and 7813 were designed (Suppl. Table 1) based on the salmon contig 107111 provided by the International Collaboration to Sequence the Atlantic Salmon Genome (ICSASG). The PCR protocol using Herculase (Agilent Technologies) consisted of the following steps: 1) an initial denaturation of 10 min at 92 °C; 2) 45 cycles of 30 sec at 92 °C, 30 sec at 58 °C, and 1 min at 68 °C. The PCR product was purified and sequenced by BigDye® Terminator v3.1 kit (Applied Biosystems). To amplify MRAP2, primer 7771 was designed based on the salmon contig 30619, while primer 7860 was based on contig 25970 (Suppl. Table 1). Both contigs were provided by ICSASG. The PCR protocol using Herculase (Agilent Technologies) consisted of the following steps: 1) an initial denaturation of 10 min at 92 °C, 2) 45 cycles of 30 sec at 92 °C, 30 sec at 58 °C, and 1 min at 68 °C. The PCR product was purified and sequenced by BigDye® Terminator v3.1 kit (Applied Biosystems).

***MC2R*:** Full length *MC2R* nucleotide is available (EU119870) in GenBank. Primers 7650 and 7651 (Suppl. Table 1) were designed to amplify the complete coding region. A previously described PCR protocol was followed [2].

Tissue specific gene expression

Quantitative gene expression was assessed by real competitive PCR (rcPCR) [3] as described by the manufacturer. The primers and competitors were designed for the standard homogenous MassEXTEND (hME) stop reaction, although iPLEX enzyme and iPLEX protocol were used for the extension PCR, as they increase the efficiency in the analysis of high multiplexes. The relative gene expression was determined using specific PCR primers to co-amplify cDNA, and competitors of known concentrations. The amplification was followed by a second extension PCR and scoring on the MassARRAY system. Furthermore, 2 technical PCR replicates and 2 printing replicates were made to ensure the reproducibility. Samples were run on four different plates with 2 fish from each of the four groups, LR *vs.* HR and spotted *vs.* non-spotted on each plate. Skin and interrenal tissue samples were collected from 8 LR and 8 HR fish, and 8 spotted and 8 non-spotted fish from a commercial fish farm in Valdres, Norway (Valdres Ørretoppdrett,

Valdres, Norway, stock is AquaGen ASA, www.aquagen.no). All these fish were non-stressed, i.e. sampled directly from their rearing tank or from social isolation. Expression of *MC1R* (paralogs 1 and 2) was measured in one multiplex using allelotyping [4] with *ACTB* and *GPDH* as housekeeping genes (for primer sequence see Suppl. Table 2). *ASIP* and *MC2R* were amplified in another multiplex (for primer sequence see Suppl. Table 3), together with the housekeeping genes *ACTB*, *GPDH*, *S18* and *RS11*. Purified total RNA was treated with TURBO DNA-free™ (Ambion) for removal of contaminating DNA. First strand cDNA synthesis was conducted using SuperScript™-II RNase H, Reverse Transcriptase (Invitrogen), oligo-dT primer T270, and 5 µg of total RNA from each tissue was used as template.

Effect of cortisol on ASIP expression
In total, 16 LR trout were isolated in 125 l compartments in glass observation aquaria. The fish were allowed to acclimate to the experimental set-up for 10 days. During acclimation they were fed pelleted trout feed (1% of body weight) once daily, between 12.00 and 16.00, by dropping pellets one by one into the aquarium. Surplus feed was removed. On day 11, 8 fish were injected with high dose of cortisol (84 mg/kg body weight) prepared in coconut oil:palm oil, (60:40) ratio, in the peritoneal cavity. Another 8 fish were injected with the vehicle solution to serve as control. After 14 days post injection, fish were anesthetized and blood samples were collected from the caudal vein. Skin and interrenal tissue samples were collected for expression analyses. The multiplex shown in Suppl. Table 3 was used as described in the paragraph above.

Correlation between MC2R, MRAP and MRAP2 expression.
The correlation of *MC2R* mRNA level with *MRAP* and *MRAP2* mRNA level in trout was analyzed by real time PCR. Head kidney tissue from 8 non-stressed HR and 8 non-stressed LR trout was collected in RNAlater. Total RNA was isolated, and qtRT-PCRs were carried out using a Roche LC480 light cycler (Roche Diagnostics). Reaction volumes were 10 µl, and included Light cycler® 480 SYBR Green I Master (Roche Diagnostics GmbH), primers (5 µM each, for primer sequence refer to Suppl. Table 4) and cDNA. Cycling conditions were as follows: 10 min at 95°C, 42 cycles of 10 sec at 95°C, 10 sec at 60°C and 10 sec at 72°C followed by melt curve analysis. All reactions were run in duplicate and controls without DNA template were included to verify the absence of contamination. Relative gene expression data was calculated from qtRT-PCR raw data using the formula:${}_{IC}E^{Cp}/{}_{GOI}E^{Cp}$= Expression of GOI in ratio to IC, where IC is internal control (*ACTB*), GOI is gene of interest, E is priming efficiency, and Cp is the crossing point. E values were calculated for each qtRT-PCR reaction using LinRegPCR software, version 11.30.0 [4].

Statistical analysis.
Since two housekeeping genes were common in both multiplexes for the real competitive PCR analysis, the data were controlled for possible multiplex effects. The correlation between *AF* and *ACTB* was 0.78 ($p<0.001$) and between *ACTB* and *GDPH* was 0.90 ($p<0.001$), indicating few multiplex effects in the estimation of the expression level. As the other housekeeping genes showed high variability between samples, expression levels were normalised using the average of *GPDH* and *ACTB*. Hence normalised expression levels were obtained as $z_{ig} = y_{ig} – nf_i$ where y_{ig} is the observed $\log_{10}$ concentration of sample *i* and gene *g*, and nf_i is the average of the $\log_{10}$ concentration of *AF* and *ACTB* for sample *i* (i=1,…,64, g=1,…,4). For each gene and tissue, a linear model including group (HR/LR and non-spotted/spotted), plate and the interaction between group and plate. More precisely the model used for both data sets was z_{ijk} = mean +

$group_j + plate_k + group_j \times plate_k + error_{ijk}$ (i=1,…,64, j=1,2, k=1,…,4). All these analyses were done using Matlab. Gene expression levels were then normalized with LR (or spotted) average = 1, and fold changes were compared statistically using t-test. The latter approach was also used for data from real time PCR, which was utilized when paralog specific analysis was not required. Behavior of spotted and non spotted fish was compared using t-test for the parameters total feed consumed (expressed as % of individual body weight) during the observation period, and time spent moving in the acute stress test. Plasma cortisol samples of spotted and non-spotted trout, stressed and non-stressed, were analyzed with two-way ANOVA (n=8 pr group) indicating an effect of stress at the level of p= 0.01 and an effect of group (spotted *vs* non-spotted) at p=0.05. Correlations were analyzed statistically by linear regression.

Supplementary results

MC1R: Sequencing of MC1R revealed two distinct variants of this gene that showed 97 % identity (100 % coverage) at the nucleotide level. We assume that these two variants represent two paralogs of the MC1R gene in rainbow trout, and the paralogs were termed MC1R_paralog1 (Accession#: FN821693) and MC1R_paralog 2 (Accession#: FN821694), respectively. Both paralogs contain an open reading frame that encodes a protein consisting of 338 amino acids. The MC1R paralogs differed only at one amino acid (aa) position. While paralog 1 has Methionine (Met) in aa-position 23, paralog 2 has Valine (Val) in the corresponding position. Alignment of the predicted full-length amino acid sequence of rainbow trout MC1R together with other known vertebrates suggests that trout MC1R has highest sequence identity with *Dicentrarchus labrax* (79 % identity, 94 % coverage). The high number of synonymous (24) compared to non-synonymous (1) nucleotide differences between the two paralogs indicates a strong selection against non-synonymous mutations. This is a bit suprising given the existence of two paralogs of this gene, and the relatively large amount of MC1R variation found in other species e.g. humans [5]. The apparent selection pressure, and the fact that the two paralogs are almost equally expressed in the experimental groups, may indicate that both paralogs are functional genes.

ASIP: The putative ASIP sequence contained an open reading frame of 122 amino acids (Accession#: FN821692). The overall amino acid sequence of trout agouti is 55 % identical to *Takifugu rubripes* (100 % coverage). No indication of any paralogous ASIP sequence was observed. There is one potential SNP (c.181A>C) observed, leading to replacement of Thr with Pro. The SNP did not follow any of the phenotypic groups.

MRAP and MRAP2: The putative MRAP sequence (FR837908) contained an open reading frame of 78 amino acids, which is shorter than reported in other organisms. The overall amino acid sequence showed 72% identity (69 % coverage) to *Meleagris gallopara* and *Gallus gallus.* MRAP2 (FR837909) contained an open reading frame of 238 amino acids. The overall amino acid sequence was 48 % identical (91 % coverage) to *Monodelphis domestica*.

MC2R: No indication of any paralogous *MC2R* sequence was observed. Several SNPs were observed, but neither followed any particular phenotypic group.

Modeling of MC1R_paralog 2

A structural model of MC1R_ paralog 2 was made using I-TASSER. The seven conserved transmembrane helices (TM1-7) are clearly visible (Figure S1). The residue displayed in ball stick corresponds to the L176M residue, and is located in the 4th transmembrane domain (TM4). Residue 176 in rainbow trout MC1R corresponds to human MC1R residue 170. This residue is Leu in most bony fishes and birds, while Val or Ala is most common in mammals. However, in mouse Met is found in the corresponding position. Both the location and the lack of conservation of residue 176 might suggest that it is not crucial in ligand binding. However, this transmembrane domain may be involved in MRAP binding in the endoplasmic reticulum and subsequent membrane trafficking [6].

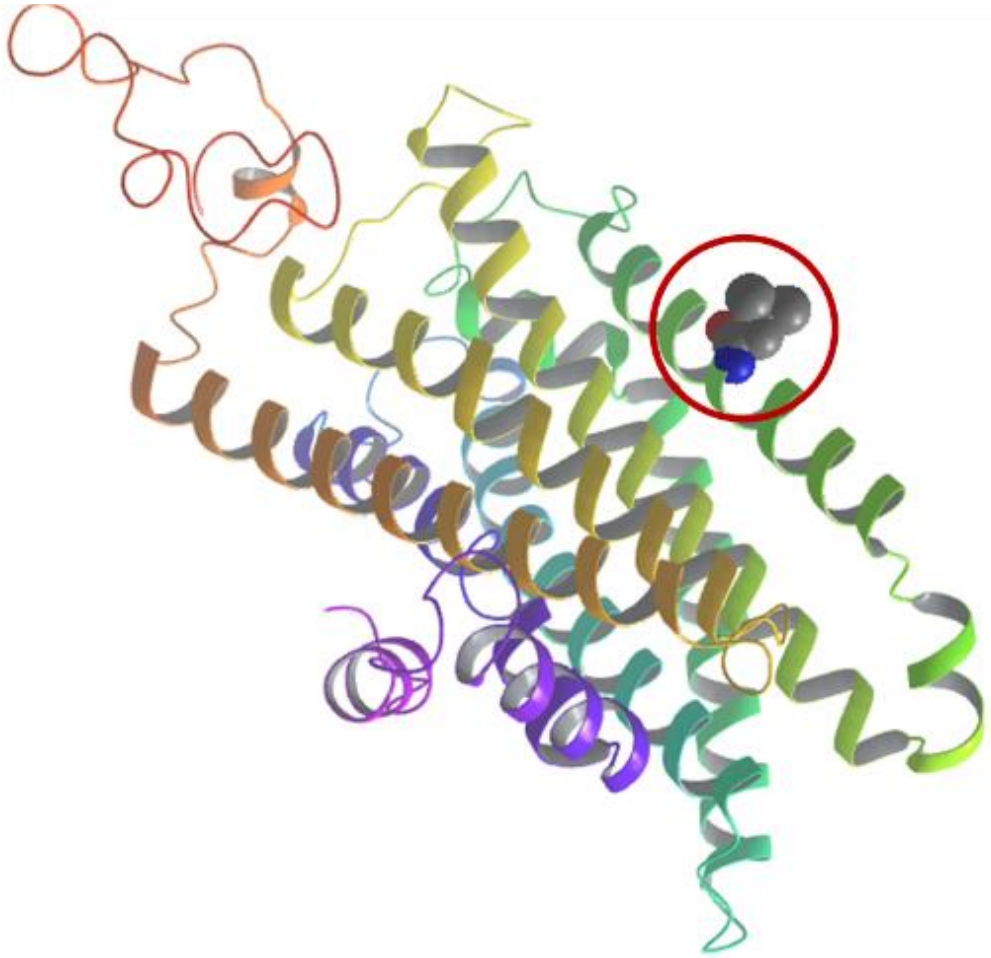

Supplementary Figure 1

The homology models obtained from I-TASSER for MC1R_paralog 2. The residue displayed in ball and stick corresponds to the L176L residue, and is located in the 4th transmembrane domain (TM4). The N terminus = orange and the C terminus = purple

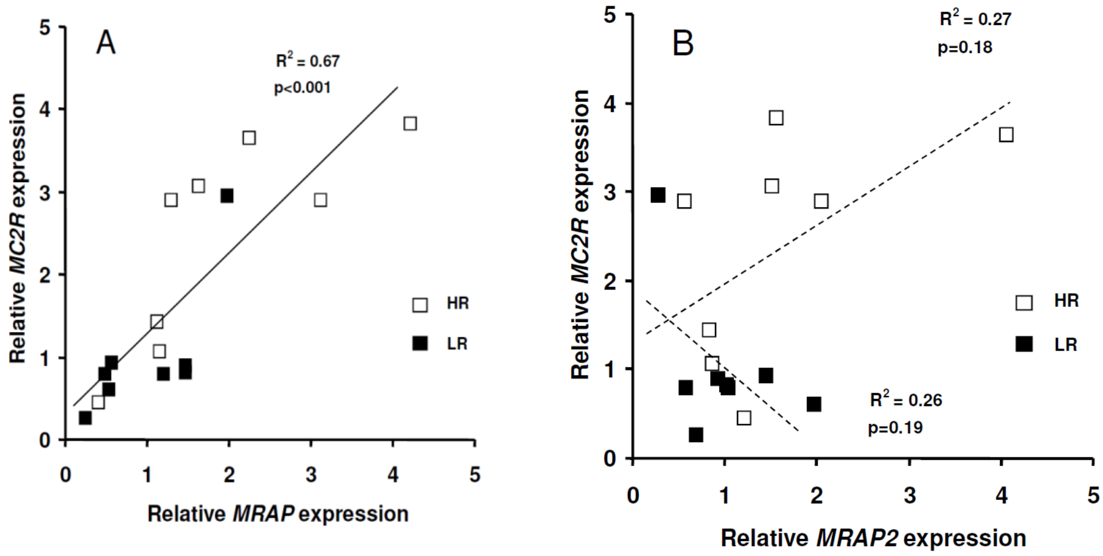

Supplementary Figure 2

Regression between the relative expression of Melanocortin 2 Receptor and (A) Melanocortin 2 Receptor Accessory Protein (*MRAP)* and (B) *MRAP2* in head kidney tissue from high- (HR) and low-responsive (LR) trout lines. Gene expression data are presented as fold change with LR average = 1. *MRAP* produced a significant common regression line with *MC2R* (difference between slopes p=0.62, deviation from linearity p=0.62). Separate regression lines were also significant for both HR and LR, with linear regression p=0.02 and p=0.03, respectively. MRAP2 showed no such relationship (two separate non-significant regression lines, difference between slopes p=0.04).

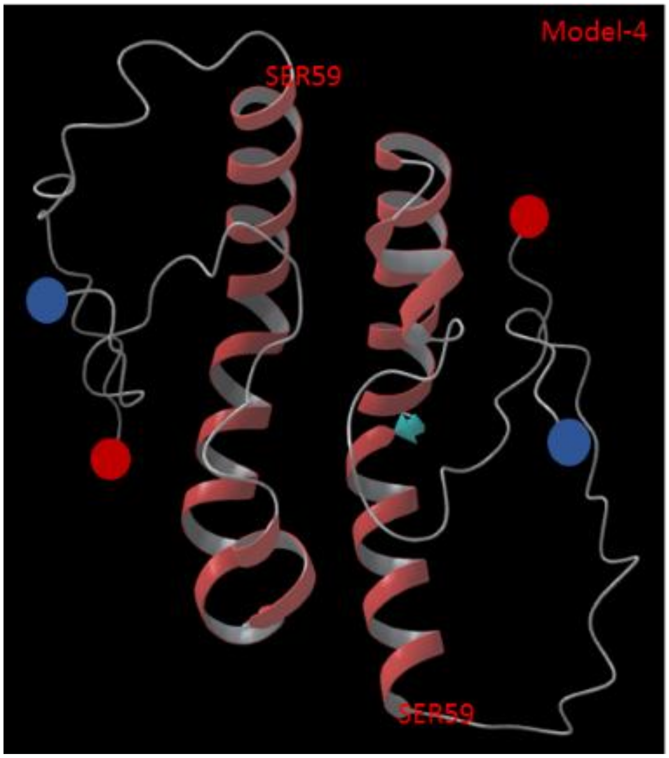

Supplementary Figure 3

Dimeric model of MRAP as obtained from ClusPro based dimerization. The N terminus = blue dot and the C terminus = red dot.

Primers	Primer code	Sequence (5' - 3')
MC1R F	7600	TGTAGAGCACTGCGGTGAAG
MC1R R	7601	CAAGGCATTTTATTTCCACCT
MC1R 5`	7613	CTGACGCTGGGCCTCATTAGTCTG
MC1R 5`	7614	ACAACGTCATCGACATCATGAACTGC
MC1R 3`	7621	CAGACTAATGAGGCCCAGCGTCAG
MC1R 3`	7622	GCAGTTCATGATGTCGATGACGTTGT
MC1R 3`	7635	TCACGCTCACTATCCTGCTAGGGGTCT
MC1R 3`	7636	GGGGACCCTTCTTCCTCCACCTTATTC
Full MC1R _par1F	7637	CGGTCACAGTCCAGTCGAAT
Full MC1R_par1 R	7641	GCAGGCATTCGCCTAATAGT
Full MC1R_par2 F	7638	CGGTCACAGTCCAGTTGAAA
Full MC1R _par2 R	7642	GGCAGGCATTCGCCTAATAGTG
Full MC2R F	7650	TGTGGACGTTGCTCTGACAC
Full MC2R R	7651	GGGTCAAAAACTCTGGTGGA
Full ASIP F	7664	CCTGCAGGAAAAAGTGGAAG
Full ASIP R	7663	CAGACATTTTCTCTCCCTTGTG
MC1R	7627	CCTCATTCAGCAGCCTATTACAGCAGA
MC1R	7628	CTCACCGTGTACAGTGAGAACAGCACT
MRAP F	7806	ACTGGGCTTCCTCTCATCAC
MRAP R	7813	GACGAAATTGAGGGCTGTTC
MRAP2 F	7771	CCTGCGCTCTTGGATCTATT
MRAP2 R	7860	AGGCTGGTGAGTCCTGTGAT

Supplementary Table 1

List of primers for amplification and sequencing of *MC1R*, *ASIP*, *MC2R*, *MRAP* and *MRAP2*

**Primer Id**	**Sequence (5’ - 3’)**
MC1R_GA_F	ACGTTGGATGTCGTAGAGTTCTGCTCCCTG
MC1R_GA_R	ACGTTGGATGTCATGCACCACCACATGGAG
ACTB_F	ACGTTGGATGTGTGCAAAGCCGGATTCGCC
ACTB_R	ACGTTGGATGCAACCATCACTCCCTGATGCC
GPDH_F	ACGTTGGATGAACGTCTCAGTGGTGGACCT
GPDH_R	ACGTTGGATGTGGCGTCGTAGCTGGCAGG
MC1R_GA_Extension primer	AGCTGAGCACCCTCACCG
ACTB_Extension primer	AGGGGAAGACAGCCCGAG
GPDH_Extension primer	TGGCAGGCTTCTCCAGACG
MC1R_GA_competitor	TCGTAGAGTTCTGCTCCCTGGCGCCGGTGTTGTTNGTGCTGTTCTCA<br>CAaGGTGAGGGTGCTCAGCTCCATGTGGTGGTGCATGA
ACTB_competitor	CAACCATCACTCCCTGATGCCTGGGACGCCCGACGATGGAGGGGAA<br>GCCCGAcGCGCGTCATCTCCGGCGAATCCGGCTTTGCACA
GPDH _competitor	TGGCGTCGTAGCTGGCAGGCTTCTCCAGACcGACGGTCAGGTCCAC[illegible]<br>AGACGTT

Supplementary Table 2

List for primers for 3 Plex MassArray used for real competitive PCR (rcPCR)

Primer Id	Sequence (5’ - 3’)
MC2R _F	ACGTTGGATGCGCTGAACTCCATCATTAGG
MC2R_R	ACGTTGGATGGTAGCCTGGCCACGTTCAACA
ASIP _F	ACGTTGGATGCGTCAAAAACAAACGTCCACC
ASIP_R	ACGTTGGATGCACAGCACACATTATTAGGG
S18_F	ACGTTGGATGCTGATCTTCTTCAGCCTCTC
S18_R	ACGTTGGATGCAAGTACAGCCAGGTCCTTGC
ACTB_F	ACGTTGGATGCCAACCATCACTCCCTGATGC
ACTB_R	ACGTTGGATGGTATGTGCAAAGCCGGATTC
RS11_F	ACGTTGGATGATACGACCACGGATGGAGAC
RS11_R	ACGTTGGATGACTCCAAGAGAGGCTATTGAT
GPDH _F	ACGTTGGATGGTCGTAGCTGGCAGGCTTCTC
GPDH _R	ACGTTGGATGATCACTGGCATGGCCTTCCG
MC2R-Extension primer	TCTCCTCCCTCTCCAAG
ASIP- Extension primer	AGCTTCCCCACAATGG
S18- Extension primer	CTGGACAACAAGCTGA
ACTB- Extension primer	GCTGTCTTCCCCTCCATC
RS11-Extension primer	TGACAAGAAATGCCCCTT
GPDH- Extension primer	CCCCCAACGTCTCAGTGGTG
MC2R-competitor	CGCTGAACTCCATCATTAGGGTTTCCCAGGaCTTGGAGAGGGAGGAGAT<br>TGAACGTGGCCAGGCTAC
ASIP-competitor	CACAGCACACATTATTAGGGGTTTTGCAGCTTCCCCACAATGGCtCGCA[illegible]<br>CGGGAGGAGGTGGACGTTTGTTTTTGACG
S18- competitor	CTGATCTTCTTCAGCCTCTCAAGATCCTCaCTCAGCTTGTTGTCCAGACC[illegible]<br>CAAGGACCTGGCTGTACTTG
ACTB- competitor	CCAACCATCACTCCCTGATGCCTGGGACGCCCGtCGATGGAGGGGAAGA<br>CCGAGGCGCGTCATCTCCGGCGAATCCGGCTTTGCACATAC
RS11-competitor	ATACGACCACGGATGGAGACATTTCCAGaGAAGGGGCATTTCTTGTCAA<br>GTGCCATCAATAGCCTCTCTTGGAG
GPDH-competitor	GTCGTAGCTGGCAGGCTTCTCCAGACGGACGGTCAGGaCCACCACTGAG<br>GGGGGTGGGGACACGGAAGGCCATGCCAGTGAT

Supplementary Table 3

List for primers for 6 Plex MassArray used for real competitive PCR (rcPCR)

Primer Id	Sequence (5’ - 3’)
MRAP_F	ACCCAATACCAGTGGACGAG
MRAP_R	TGAACGGGACATGAAAGTCA
MRAP2_F	GTTTCGTTCGAGGGACTGAA
MRAP2_R	GGAGCTCCTGTCTTGGTGAG
MC2R_F	GTGCTACCGCTCTCTGTTCC
MC2R_R	GGAGCAGAACAGCATCTTCC

Supplementary Table 4

List of primers for real-time PCR.

References

1 Pottinger, T. G. & Carrick, T. R. Modification of the plasma cortisol response to stress in rainbow trout by selective breeding. *General and comparative endocrinology* **116**, 122-132 (1999).

2 Aluru, N. & Vijayan, M. M. Molecular characterization, tissue-specific expression, and regulation of melanocortin 2 receptor in rainbow trout. *Endocrinology* **149**, 4577-4588, doi:10.1210/en.2008-0435 (2008).

3 Ding, C. & Cantor, C. R. A high-throughput gene expression analysis technique using competitive PCR and matrix-assisted laser desorption ionization time-of-flight MS. *Proceedings of the National Academy of Sciences of the United States of America* **100**, 3059-3064, doi:10.1073/pnas.0630494100 (2003).

4 Ruijter, J. M. *et al.* Amplification efficiency: linking baseline and bias in the analysis of quantitative PCR data. *Nucleic acids research* **37**, e45, doi:10.1093/nar/gkp045 (2009).

5 Gerstenblith, M. R., Goldstein, A. M., Fargnoli, M. C., Peris, K. & Landi, M. T. Comprehensive evaluation of allele frequency differences of MC1R variants across populations. *Hum Mutat* **28**, 495-505, doi:10.1002/humu.20476 [doi] (2007).

6 Chan, L. F. *et al.* MRAP and MRAP2 are bidirectional regulators of the melanocortin receptor family. *Proceedings of the National Academy of Sciences of the United States of America* **106**, 6146-6151, doi:10.1073/pnas.0809918106 (2009).
